# Incidence and Importance of Peripheral Vestibular Dysfunction in Adolescent Idiopathic Scoliosis

**DOI:** 10.3390/children11060723

**Published:** 2024-06-13

**Authors:** Liliana Vlădăreanu, Mădălina Gabriela Iliescu, Iulia Tania Andronache, Elena Danteș

**Affiliations:** 1 Doctoral School of Medicine, “Ovidius” University of Constanta, 1 University Alley, 900470 Constanta, Romania; liliana.vladareanu@sbtghiol.ro (L.V.); andronacheiulia@gmail.com (I.T.A.); elena.dantes@365.univ-ovidius.ro (E.D.); 2 Rehabilitation Department, Faculty of Medicine, “Ovidius” University of Constanta, 1 University Alley, 900470 Constanta, Romania; 3 Pediatric Neurorehabilitation Department, Techirghiol Balneal and Rehabilitation Sanatorium, 34-41 Climescu Blvd., 906100 Techirghiol, Romania; 4 Department of Rheumatology, Internal Medicine Clinic, “Alexandru Gafencu” Military Emergency Hospital Constanta, Mamaia Blvd., 900527 Constanța, Romania; 5 Clinical Hospital of Pulmonology, 40 Sentinelei Street, 900002 Constanta, Romania

**Keywords:** adolescent idiopathic scoliosis, peripheral vestibular dysfunction, vestibular deficit, Fukuda stepping test, motion sickness

## Abstract

Adolescent idiopathic scoliosis (AIS) is a common form of scoliosis. As the name suggests, etiopathogenesis is not clearly defined, so treatment is still anchored in the musculoskeletal theory and correction/prevention of high Cobb angle values. This study aimed to determine whether there is any connection between developing scoliotic curvature and a positive history of motion sickness as a symptom of a peripheral vestibular dysfunction/deficit, and if vestibular rehabilitation exercises could be integrated into the treatment plan. The study was conducted over 12 months on a selected population of 159 patients to evaluate or treat scoliotic curvatures in a private clinic. The collected data were analyzed using IBM SPSS Statistics 25 and illustrated using Microsoft Office Excel/Word 2021. Patients with peripheral vestibular dysfunction had significantly higher Cobb angle values when compared to patients with a negative result in an instrumental test for peripheral vestibular dysfunction. Motion sickness was considerably more associated with peripheral vestibular dysfunction, and a positive Fukuda stepping test was associated with a positive history of motion sickness. Adolescent idiopathic scoliosis with higher Cobb angles is related to positive motion sickness history as part of peripheral vestibular dysfunction. Conservative treatment for scoliosis could incorporate sensory integration techniques, and a positive history of motion sickness could be an indicator of a higher risk of progression in adolescent idiopathic scoliosis.

## 1. Introduction

Adolescent idiopathic scoliosis (AIS) is defined by the appearance of non-physiological curves in the frontal plane of the spine, regardless of location, with unknown etiological cause, and with a Cobb angle value that must exceed 10°. The onset age of adolescent idiopathic scoliosis is 9–10 years for girls and 11–12 years for boys. Adolescent idiopathic scoliosis (AIS) is the most common form of scoliosis, with a global prevalence ranging from 2 to 3% to 5.2% [1,2,3,4,5]. AIS is classified according to the Cobb angle value and the location and orientation of the pathological curve [6,7,8,9].

Even if the latest research mentions probable genetic and neuroendocrine factors as etiopathogenic causes, the treatment of adolescent scoliosis remains anchored in theories of human biomechanics, with two main types: non-surgical (conservative) and surgical. Conservative treatment is aimed at scoliosis with Cobb angles below 45°, with a slowly progressive or stationary evolution over time, involving specific kinetic techniques—physiotherapy scoliosis-specific exercise (PSSE), scientific exercise approach to scoliosis (SEAS), Schroth, Rigo, etc.—and specific braces (Cheneau, Gensingen, etc.). Surgical treatment is recommended for scoliosis that exceeds the Cobb value of 45°–50° associated with a Risser index above three or rapidly progressive scoliosis (over 10° Cobb angle progression in 3 months with or without appropriate conservative treatment) [10,11,12,13,14,15]. It involves different techniques, depending on the patient’s age and clinical status.

One of the neurological theories that has been researched lately, but not wholly proven, implies that there is a connection between a faulty vestibular system and scoliotic curvature, meaning that patients with AIS are more likely to have vestibular peripheral dysfunction compared to patients from control groups [16,17,18,19,20,21,22,23,24]. The vestibular system influences the sense of orientation, stational or in movement, and the perception of the vertical, right–left, and rotational direction, etc. [25,26,27,28,29,30,31,32]. Suppose the perception of the vertical is inaccurate from the start of motor acquisition or later motor development due to a peripheral vestibular dysfunction. Is it possible to maintain a straight spine, with no lateral inclination or axial rotation as in AIS, during rapid skeletal growth?

Motion sickness is already related to a sensitive vestibular system, unilateral or bilateral, manifesting from as early as one year of age. If the child is under six years of age, the main symptom is dizziness; later in life, the main symptom is nausea, but one does not exclude the other. Another question is whether motion sickness, as a symptom of peripheral vestibular dysfunction, can be a potential predictor of a higher risk of scoliotic curvature development and a higher Cobb value over time.

The stepping test is a simple bedside clinical test developed by Unterberger in 1938, and then refined and popularized by Fukuda in 1959 to evaluate vestibular dysfunction [33,34,35]. It consists of the patient, in an upright position, with eyes closed, stepping in place for 50 to 100 paces. It is considered positive if the patient rotates more than 45° to the right or to the left and the rotation is towards the affected side [33,34,35] The rotation is directly proportional to the intensity of the symptoms for peripheral vestibular dysfunction and the test is more relevant if the symptoms are more severe [33,34,35].

Considering that currently there is no association between scoliotic screening and a history of motion sickness or vestibular dysfunction, we decided to check if, among the children presented in the clinic for the evaluation and treatment of scoliotic curvatures, there was a significant number with a history of motion sickness and a positive Fukuda stepping test (FST)—the most common clinical sign for vestibular dysfunction—and whether the results of their radiographs showed any possible connection between Cobb angle values, their personal history of motion sickness, and/or a positive FST.

## 2. Materials and Methods

### 2.1. Ethical Approval

Written informed consent for the procedures and use of the data was obtained from all participants, either from just the patients (for those aged 16 and above) or from the patients and their parents (for those below the age of 16). Ethical approval from the medical unit where this paper was conceived was obtained with ethical form no. 3739/15 March 2022.

### 2.2. Subjects

Out of 443 patients evaluated in the clinic over 12 months, we selected a final total of 159 patients’ data, from 93 females and 66 males, ages 7 to 18. They were present in the clinic over 12 months to evaluate and treat developmental vertebral disorders. The patients had to be capable of independent gait to perform the Fukuda stepping test.

### 2.3. Methodology

The data of the patients presented in the clinic and consulted over one year were analyzed in a retrospective observational study. Out of 443 patients, adults and children, evaluated during that period, only 159 patients were selected, following the inclusion criteria described below.

#### 2.3.1. Inclusion Criteria

- Age under 18 years;
- Primary vertebral disorders as the main diagnostic;
- Independent ambulation;
- Written consent for the use of personal data for scientific analysis.

#### 2.3.2. Exclusion Criteria

- Age above 18 years;
- Personal positive history for neurological, congenital genetic, or orthopedic pathology;
- Assisted ambulation;
- Lack of written consent for scientific personal data analysis.

#### 2.3.3. Features of the Clinical Standard Evaluation of These Patients

- Adam’s test standing and seated [36];
- Finger–toes index (FTI) standing and supine, measured in centimeters (cm) [37];
- Fukuda stepping test (FST) [33,34,35].

The FST was conducted with the patients in a quiet room away from noise or other disturbances. The examiner explained prior to testing that the patient would step in place for 50 paces with their eyes closed, using the graded floor in the examination room as a starting point. The examiner also showed the test to the patient to ensure that it was clearly understood. When or if the patient opened their eyes during the examination, they were stopped and asked to retake the test after the examiner made sure they understood that the eyes had to stay closed during testing.

Only a positive FST from the clinical examination was considered for this paper. When the FST was positive, meaning that there was a rotational deviation bigger than 45°, the patients underwent an instrumental vestibular test on the clinic’s Multitest-Equilibre Framiral Platform. Using specific software, the platform provided their positive or negative instrumental diagnosis for peripheral vestibular syndrome.

#### 2.3.4. The Anamnestic Standard Evaluation Included the Following Questions

- Motion sickness history (positive or negative) based on a Barany Society consensus diagnosis criteria for motion sickness [38];
- Dioptric correction (emmetrope, myopic, hypermetropic, astigmatism);
- History of other pathologies.

#### 2.3.5. Imagistic Evaluation

Gold standard standing spine stitching radiograph, posterior–anterior, and lateral incidences were included, as per the SOSORT guidelines [7], with the calculation/evaluation of the following:

- Cobb angle, measured in degrees (°) (above 10° for scoliosis);
- Nash–Moe rotational quota, measured from 0 to 4;
- Risser Index measuring the iliac crest osseous maturation, from 0 to 5;
- Iliac crest asymmetry, measured in millimeters (mm);
- Spinal congenital osseous malformation.

### 2.4. Data Analysis Methodology

All the data from the study were analyzed using IBM SPSS Statistics 25 and illustrated using Microsoft Office Excel/Word 2021. Quantitative variables were tested for normal distribution using the Shapiro–Wilk Test and were written as averages with standard deviations or medians with interquartile ranges. Qualitative variables were written as counts or percentages, and differences between groups were tested using Fisher’s Exact test. Measures of associations were quantified as odds ratios with 95% confidence intervals. When applied, the performance of diagnostic tests was quantified as sensibilities, specificities, positive and negative predictive values, and accuracies, with 95% confidence intervals.

Quantitative independent variables with non-parametric distribution were tested between groups using Mann–Whitney U tests. Using the Cobb angle, an ROC curve was used to predict the diagnosis of the peripheral vestibular syndrome. The prediction performance was calculated as the AUC value with a 95% confidence interval. Based on the ROC curve, a cut-off value was obtained based on the highest value of the Youden index and its diagnostic sensibility and specificity.

## 3. Results

### 3.1. Statistical Characteristics of the Studied Batch

An analysis of the study cohort shows the following characteristics: most of the analyzed patients were girls (58.5%), with an average age in the lot of 11.54 ± 2.57 years and a median age of 11 years.

Of the 159 patients enrolled in the retrospective study, 116 consented to a gold-standard radiograph to evaluate the Cobb angle, Nash–Moe grading, and Risser score. The average Cobb angle value was 12.87 ± 5.37 degrees, with a median of 12. According to the Cobb angle classification, 76.7% (89) of the patients had scoliosis, most having grade I scoliosis (94.4%) (Table 1).

According to the Nash–Moe grading, most patients from the radiographed lot (116) had less than 25% vertebral rotation, meaning that they were Nash–Moe I (53.4%).

According to the Risser Index, most of the radiographed patients had between 50 and 75% iliac crest ossification (Risser 3) (46.6%) or between 25 and 50% ossification (Risser 2) (27.6%).

Of 159 clinically examined patients, 12.6% had a positive result for the Fukuda stepping test, and 20.8% had a history of motion sickness (Table 2).

### 3.2. Main Analyzed Hypotheses

The collected data from June 2022 to June 2023 were analyzed concerning three main hypotheses, as follows:

- Connection between Cobb angle value and peripheral vestibular dysfunction;
- Connection between positive history of motion sickness and peripheral vestibular dysfunction;
- Connection between positive history of motion sickness and Fukuda stepping test.

#### 3.2.1. Connection between Cobb Angle Value and Peripheral Vestibular Dysfunction

**Figure 1:**
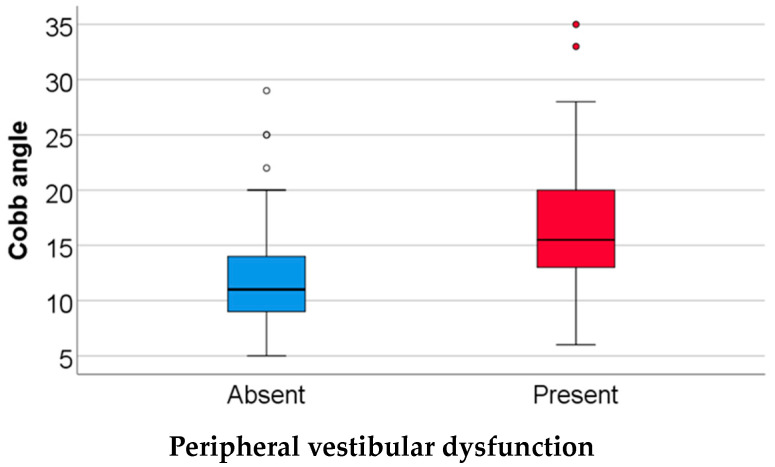
Comparison of Cobb angle according to peripheral vestibular dysfunction.

Data from Table 3 and Figure 1 show the comparison of the Cobb angle according to the existence of peripheral vestibular dysfunction instrumental diagnosis. The distribution of the Cobb angle was non-parametric in both groups, according to the Shapiro–Wilk test (*p* < 0.05).

According to the Mann–Whitney U test, differences between groups were statistically significant (*p* < 0.001), with patients with peripheral vestibular dysfunction having a significantly higher Cobb angle value (median = 15.5, IQR = 12.75–21.75) in comparison to patients without a positive result in an instrumental test for peripheral vestibular dysfunction (median = 11, IQR = 9–14).

**Figure 2:**
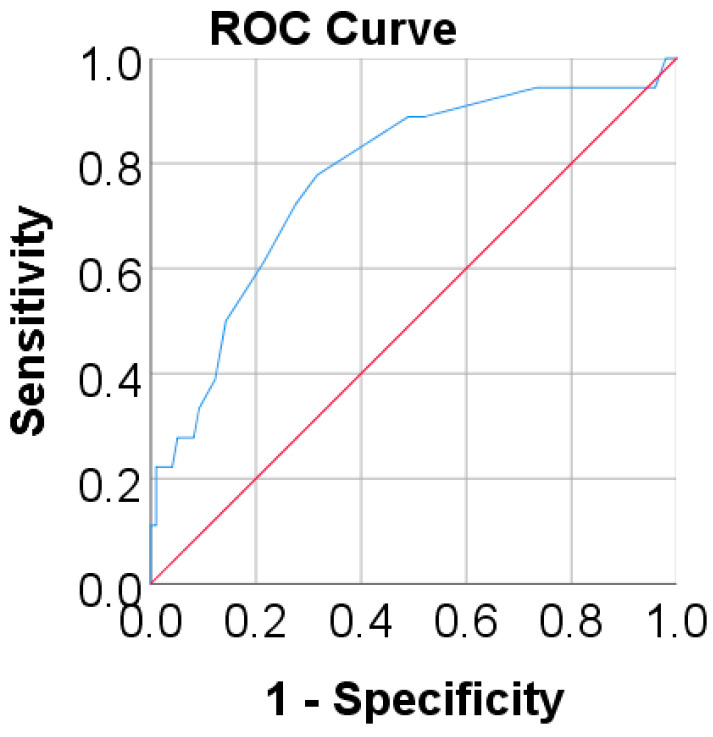
ROC curve graph using the Cobb angle to predict peripheral vestibular dysfunction.

Table 4 and Figure 2 show the ROC curve analysis for predicting peripheral vestibular dysfunction using the Cobb angle. According to the study, the performance of the prediction using the Cobb angle was significant and acceptable (*p* < 0.001, AUC = 0.774 (95% C.I.: 0.652–0.896)) and the cut-off obtained using the highest value of the Youden index (J = 0.461) was 12.5; as such, a test using a value equal or higher than 12.5 for Cobb angle has a 77.8% sensitivity and 68.4% specificity for the diagnosis of peripheral vestibular dysfunction.

#### 3.2.2. Connection of Motion Sickness History and Peripheral Vestibular Dysfunction

**Figure 3:**
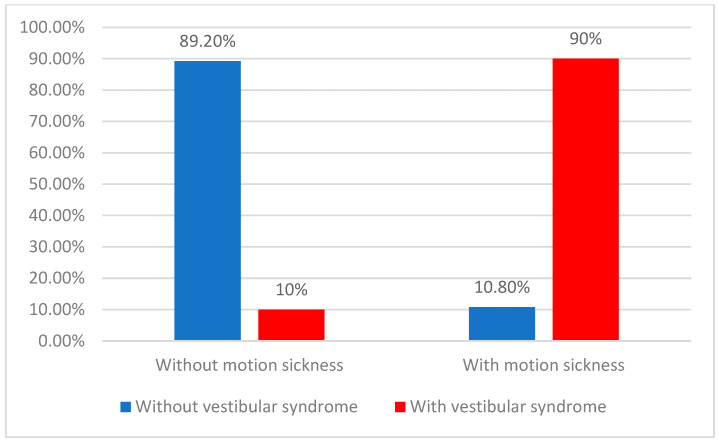
Distribution of patients according to motion sickness positive history and a positive instrumental diagnosis of peripheral vestibular dysfunction.

Data from Table 5 and Figure 3 show the distribution of patients according to the existence of motion sickness and an instrumental diagnosis of peripheral vestibular dysfunction. Differences between groups were statistically significant according to the Fisher’s Exact Test (*p* < 0.001); patients with motion sickness were significantly more associated with peripheral vestibular dysfunction (90% vs. 10.8%), and patients with motion sickness had increased odds of having peripheral vestibular dysfunction by 74.4 times (95% C.I.: 15.696–352.652).

#### 3.2.3. Correlation between Positive History of Motion Sickness and Positive FST

**Figure 4:**
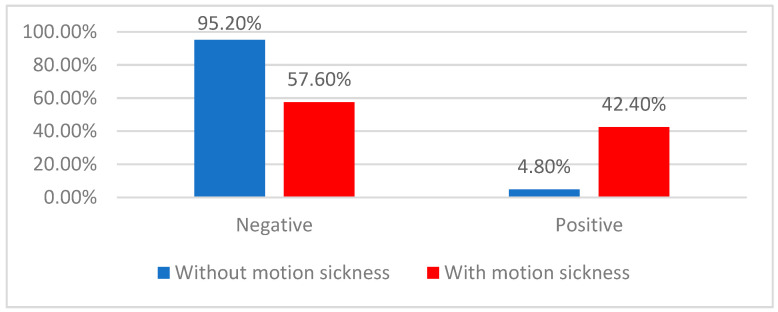
Distribution of the patients according to the Fukuda test and history of motion sickness.

Data from Table 6 and Figure 4 show the distribution of the patients according to a positive Fukuda stepping test and the existence of a history of motion sickness. Differences between groups were statistically significant according to the Fisher’s Exact Test (*p* < 0.001); patients with a positive Fukuda stepping test were significantly more associated with a positive history for motion sickness (42.4% vs. 4.8%), with patients with a positive Fukuda test having increased odds of having motion sickness, by 14.737 times (95% C.I.: 5.046–43.042).

In this scenario, if the Fukuda stepping test were used for the diagnosis of motion sickness, it would have a sensitivity of 42.42% (95% C.I.: 25.48–60.78), specificity of 95.24% (95% C.I.:89.92–98.23), a positive predictive value of 70% (95% C.I.: 49.28–84.86), and a negative predictive value of 86.33% (95% C.I.: 82.46–89.46), overall having an accuracy of 84.28% (95% C.I.: 77.67–89.56).

## 4. Discussion

Given its multifactorial features, there is no conclusive evidence of a single specific biomarker used to precisely diagnose and measure the risk of progression in AIS which is also widely available outside of research laboratories. We have focused our research on identifying the relationship between measurable radiographical parameters, such as Cobb angle, Risser index, Nash–Moe rotational quotation, and iliac crest asymmetry, as per SOSORT guidelines, and the positive history of motion sickness or vestibular pathology—namely peripheral vestibular dysfunction—through a specific clinical test, the FST. In this batch, most of the patients with scoliotic curvatures had motion sickness history, suggesting the potential use of this positive history as a predictive instrument for developmental scoliotic curves and their evolution, with the possible result of early intervention for these patients, which could save them, their families, and society from the high economical and psychological cost of bracing and/or spine surgery.

### 4.1. Comparison of Published Data

The main objective of this retrospective study was to determine whether the patients who presented themselves in the clinic for the evaluation and treatment of spinal deformities that were classified as adolescent idiopathic scoliosis had any peripheral vestibular dysfunction as well, and if this deficit, which was determined clinically by a positive FST, was in any way related to the Cobb angle value on the radiograph. As secondary objectives, we tried to determine whether a positive motion sickness history can be linked to an instrumental diagnosis of peripheral vestibular dysfunction and whether a connection between a positive FST and a positive history of motion sickness can be made.

Vestibular dysfunction is a disturbance/modification of the body’s balance system. It can be central or peripheral. In some of the studies conducted in the past to determine the etiopathogenesis of AIS, it was demonstrated that there is a direct relationship between functional and anatomical deviations of the vestibular organ and a scoliotic change [16,17,18,19,20,21,22,23,24]. Causes and explanations differ considerably. There is significant debate regarding the possible causes and explanations; therefore, it is essential to understand and differentiate whether faulty vestibular information in AIS patients arises due to vestibular organ malformation or during the processing of the vestibular afferents at cortical and subcortical levels [16,17,18,19,20,21,22,23,24].

As mentioned, vestibular dysfunction can be peripheral or central [25,26,27,28,29,30,31,32].

A central vestibular syndrome can be related to a demyelinating disease (e.g., Multiple Sclerosis), a genetic disease (e.g., Friedreich Ataxia), a cerebellar stroke, etc., that will result in a lesion situated in the vestibular nuclei or any of their central projections, especially those from the cerebellum [25,26,27,28,29]. Peripheral vestibular syndrome usually presents acutely, with the most prevalent forms of acute peripheral vestibular dysfunction being benign paroxysmal positional vertigo (BPPV), ear infection, etc. [27]. A cluster of symptoms present for both central and peripheral vestibular syndrome, such as motion sickness, vertigo, nausea and vomiting, intolerance to head motion, spontaneous nystagmus, unsteady gait, and postural instability. They can be present together or independently and have varying degrees of intensity, with none considered pathognomonic. The most common test for diagnosing and evaluating peripheral vestibular dysfunction is the Fukuda stepping test or Unterberger stepping test (FST), which consists of walking in place for 60 s while blindfolded. It is an easy-to-perform test with good reliability that does not need special training for the evaluator or unique instrumentation in the office [33,34,35].

Our data showed that a connection could be made for this study cohort between Cobb angle value and the instrumental positive diagnosis of peripheral vestibular dysfunction (see Section 3.1), aligning our results with the literature already published [16,17,18,19,20,21,22,23,24].

### 4.2. Potential Use in Treatment

Currently, there is no real consensus about the pathogenesis of AIS [11,12,13,14,15]. Therefore, the only conservative treatment generally accepted is the one anchored in biomechanical theory [10]. This treatment, as previously mentioned, mainly relies on PSSE (physiotherapy scoliosis-specific exercises)/SEAS (scientific exercise approach to scoliosis) focused on the patient and individual bracing [10,39,40,41,42,43,44], even if there are other less promoted or studied conservative biomechanical treatments. One of the conservative biomechanical treatments less used in Europe but not without value is Chiropractic BioPhysics, shown to reduce Cobb angles when used in the treatment of scoliotic spine [45,46].

Patient outcomes are related to Cobb angle variations, meaning that patients need to take full spine radiographs at a 6-month interval for the Cobb angle, Nash–Moe quotation, and Risser index to be determined [7,8].

Extended periods of conservative treatment, spanning months to even years, can be time-consuming and financially burdensome. Hence, patients and their families frequently abandon this approach. The demanding nature of such a therapeutic strategy requires a constant awareness of muscle activity on behalf of the patient, along with the necessity to wear the brace for at least 18 h per day and to maintain active correction while wearing it. Non-adherence to conservative treatment results in an elevated Cobb angle value and Nash–Moe rotational quota, which subsequently increases the patients’ risk of surgery or development of chronic pain and functional disability [47,48].

At the same time, specific treatments have proved efficient for patients suffering from peripheral vestibular dysfunction [49,50]. This may help AIS patients with vestibular dysfunction maintain their active correction more efficiently and better orient themselves in space. Suppose this type of treatment, instrumental or classical, is used in AIS patients. In that case, the time they spend in a brace will be less, and patients will be more compliant with the treatment and more able to maintain muscle active correction during bracing.

The current non-surgical treatment consensus for adolescent idiopathic scoliosis states that physical exercise should be patient-centered and that bracing should be personalized. The exercises aim to decrease the rotation and lateral inclination of the spine using training in corrected positions in activities of daily life [10]. In the up-to-date protocol, there is no mention of complex sensorial integration—vestibular, visual, proprioceptive—even if there is proof that scoliotic patients suffer from sensory integration deficits, making them less aware of their antigravitational muscular tonus. While this hypothesis requires further investigation, it should not be disregarded. These exercises and non-surgical treatments could optimize the outcomes, possibly reducing bracing time or rehabilitation for patients with AIS.

### 4.3. Potential Use in Early Diagnosis

The results in the studied lot show that the higher the value of the Cobb angle on the radiograph, the higher the chances of the patient having a positive history of motion sickness and a positive FST result.

There has been no research carried out until now, as far as we know, to determine the connection between a positive FST and the progression of curvature in AIS, but there are some studies that have tried to link the type of scoliotic curvature and the FST [16,17,18,19,20,21,22,23,24]. The actual consensus about the curvature progression for AIS patients is related to the Cobb angle value and the Risser Index value, such as that the higher the initial Cobb, the higher the risk of progression, and the lower the Risser Index, the higher the risk of progression [7,8,10]. The combined risk is the highest when the patient has a high Cobb with a low Risser due to bone immaturity. As mentioned before, the radiograph is the gold standard for evaluation in AIS. Still, it exposes the patients to radiation, and it is not easily accessible in small towns, so it is necessary for the patient to travel to a big clinic, meaning that it takes more time for the correct treatment to begin [7,8,10].

Also, Europe lacks highly specialized personnel to diagnose and treat scoliosis, so correct treatment becomes even less accessible to the patient, as most of these personnel are concentrated in big cities. Medical personnel are trained to identify pathological curvatures of the spine early using simple clinical testing, such as the Adam test, and instrumentation, such as scoliometers. Still, the critical question remains: at what stage in a person’s life should this detection occur/screening start? Motion sickness appears early in life in most cases, at age two, mainly, years before the first signs of adolescent idiopathic scoliosis can be detected [7,8,10]. In the event that this hypothesis will later be sustained by other, more relevant studies, it could raise the possibility of identifying children with a higher risk of developing a scoliotic curvature and implementing simple, early interventions such as specific vestibular exercises for alleviating motion sickness [51] or integrating the oculi-vestibular reflex to potentially prevent the progression of scoliosis in these individuals [52,53,54].

Informing general practitioners, pediatricians, and parents of these at-risk children about these potential musculoskeletal issues could prompt them to refer earlier or more often to a specialist or for a simple check-up for scoliotic curvature.

### 4.4. Study Limitations and Ideas for Future Research

Our study has some limitations, such as the following: it was carried out on a small scale, so we cannot, at this moment, infer causation from correlation for a scoliotic curvature being as specific as appearing if the patient has a positive history of motion sickness or a positive FST, both as a manifestation of a peripheral vestibular disturbance/hypofunction. Also, we cannot establish without a doubt that all high-value Cobb scoliosis cases can be positively diagnosed with peripheral vestibular deficit/hypofunction. Another limitation is related to the fact that the validity of clinical testing is often dependent on the examiner’s experience, so it cannot always be objective. The anamnestic evaluation also depends, on the one hand, on the examiner’s expertise in extracting valid information from the patient and, on the other hand, on the patient’s cognitive status or potential language barriers. All of the above-mentioned limitations, in turn, add a risk of bias and/or subjectivity to the study.

However, it would be interesting to conduct a prospective study on a more numerous population and for a more extended period to determine both the incidence of motion sickness in the younger population, aged below three years, and to check what percentage—if any—of this population develops a scoliotic curvature over time and if there is any correlation/causation between the gravity of the pathological curvature and the onset of motion sickness, or if in two different lots, both with motion sickness, children treated for sensorial integration still develop or do not develop scoliotic curvatures.

Authors/researchers should discuss the results and how they can be interpreted from the perspective of previous studies and working hypotheses. The findings and their implications should be addressed in the broadest context possible. Future research directions may also be highlighted.

## 5. Conclusions

In our study cohort, there was a significantly bigger Cobb value for patients with a positive history of motion sickness and a positive Fukuda stepping test. These findings align with the already-published literature concerning the connection between the vestibular system and scoliotic curvature. Despite the growing number of published papers on the topic, a definitive consensus about the temporal relationship between scoliotic curvature and vestibular disturbance is yet to be reached.

The screening and diagnosis of adolescent scoliosis rely on inspection (Adam’s bending test), instrumentation (coulometers), and imaging (stitching spine radiograph). No specific test can predict patient outcomes, so early diagnosis is essential. The age of onset for motion sickness is around two years, while for adolescent idiopathic scoliosis, it is 9–10 years. In our lot, most patients with scoliotic curvature had a motion sickness history, suggesting the potential use of this positive history as a predictive instrument for developing scoliotic curves and their evolution. Therefore, this might have further implications for screening and early detection.

More research needs to be carried out on the subject, both retrospective and prospective, multicentered, and on a more significant number of patients, to further investigate and potentially validate this hypothesis.

## 6. Patents

No patents are pending for this retrospective research/method.

## Figures and Tables

**Table 1:** Distribution of scoliotic patients in the batch by Cobb angle value (°), Nash–Moe Index, and Risser Index.

Scoliosis Grading by Cobb Angle (>10°)			Nr. (89)	Percentage (%)
Grade I (10°–20°)			84	94.4%
Grade II (21°–30°)			5	5.6%
Grade III (31°–40°)			0	0%
Grade IV (>40°)			0	0%
Nash–Moe grading	Nr. (89)	Percentage (%)	Nr. (116)	Percentage (%)
0 1			23 62	19.8% 53.4%
2			25	21.6%
3			6	5.2%
4			0	0%
Scoliosis grading by Risser			Nr. (116)	Percentage (%)
0			2	1.7%
1			5	4.3%
2			32	27.6%
3			54	46.6%
5			23	19.2%

**Table 2:** Fukuda stepping test distribution.

Fukuda Stepping Test	Nr. (159)	Percentage (%)
Positive	20	12.6%
Negative	139	87.4%

**Table 3:** Comparison of Cobb angle according to peripheral vestibular dysfunction.

Peripheral Vestibular Dysfunction	Average ± SD	Median (IQR)	Mean Rank	*p* *
Absent (*p* < 0.001 **)	11.96 ± 4.23	11 (9–14)	53.57	<0.001
Present (*p* = 0.033 **)	17.83 ± 7.90	15.5 (12.75–21.75)	85.33	

* Mann–Whitney U test, ** Shapiro–Wilk Test.

**Table 4:** ROC curve analysis for predicting peripheral vestibular syndrome using the Cobb angle.

Parameter	AUC (95% C.I.)	Std. Error	*p* *
Cobb angle	0.774 (0.652–0.896)	0.062	<0.001

* Mann–Whitney U test.

**Table 5:** Distribution of patients according to motion sickness and peripheral vestibular dysfunction.

Motion Sickness/Peripheral Vestibular Dysfunction	No Peripheral Vestibular Dysfunction (nr)	No Peripheral Vestibular Dysfunction (%)	With Peripheral Vestibular Dysfunction (nr)	With Peripheral Vestibular Dysfunction (%)	*p* *
With motion sickness	124	89.2%	2	10%	<0.001
Without motion sickness	15	10.8%	18	90%	

* Fisher’s exact test.

**Table 6:** Distribution of the patients according to the Fukuda test and history of motion sickness.

Fukuda Test/ Motion Sickness	Without Motion Sickness (nr)	Without Motion Sickness (%)	With Motion Sickness (nr)	With Motion Sickness (%)	*p* *
Negative	120	95.2%	19	57.6%	<0.001
Positive	6	4.8%	14	42.4%	

* Fisher’s exact test.

## Data Availability

The data are part of an ongoing doctoral study, available for publication upon the completion of the thesis, and are available now in the archives of the private clinic where the study was conducted, available upon request.
